# Supplementary material for: Heterogeneity of Associations between Total and Types of Fish Intake and the Incidence of Type 2 Diabetes: Federated Meta-Analysis of 28 Prospective Studies Including 956,122 Participants
Source: Nutrients. 2021 Apr 7;13(4):1223. doi: 10.3390/nu13041223 (PMC8068031; doi:10.3390/nu13041223)
Supplement: Supplementary file 1 [file nutrients-13-01223-s001.pdf]

Supplementary Materials

Heterogeneity of associations between total and types of fish intake and the incidence of type 2 diabetes: federated meta-analysis of 29 prospective studies including 956,122 participants

Tables and Figures

Table S1. Characteristics of the 28 contributing cohort studies in the InterConnect project on the association between fish intake and type 2 diabetes.
Table S2. Dietary assessment methods and fish items available in the cohort studies participating in the InterConnect project on the association between fish intake and type 2 diabetes
Table S3. Assessment of confounders in the cohort studies participating in the InterConnect project on the association between fish intake and type 2 diabetes.
Table S4. Incidence Rate Ratio of type 2 diabetes for an extra 100g/week of fish intake in the subset of studies adjusting for: a) Family history of diabetes b) Waist circumference c) Fish oil supplements (InterConnect project on the association between fish intake and type 2 diabetes).

Figure S1. Incidence rate ratios and 95% confidence intervals for the association between the consumption of total fish (per 100g/day) and incident type 2 diabetes (secondary outcome) in InterConnect men. Model 1 (upper panel) adjusted for age, education, smoking, physical activity, alcohol intake, BMI, co-morbidities at baseline. Model 2 (lower panel) was additionally adjusted for dietary factors: energy intake, fibre, red and processed meat, fruit, vegetables, sugary drinks.
ARIC, Atherosclerosis Risk in Communities; ELSA Brasil, Brazilian Longitudinal Study of Adult Health; CARDIA, Coronary Artery Risk Development in Young Adults Study; MESA, Multi-Ethnic Study of Atherosclerosis; PRHHP, Puerto Rico Heart Health Program; FMC, Finnish Mobile Clinic Health Examination Survey; COSM, Cohort of Swedish Men; SUN, Seguimiento Universidad de Navarra (University of Navarra Follow-up); AusDiab, Australian Diabetes, Obesity and Lifestyle Study; CKB, China Kadoorie Biobank; JPHC, Japan Public Health Center-based; NHAPC, Nutrition and Health of Aging Population of China Study; SMHS, Shanghai Men Health Study.

Figure S2. Incidence rate ratios and 95% confidence intervals for the association between the consumption of total fish (per 100g/day) and incident type 2 diabetes (secondary outcome) in InterConnect women. Model 1 (upper panel) adjusted for age, education, smoking, physical activity, alcohol intake, BMI, co-morbidities at baseline. Model 2 (lower panel) was additionally adjusted for dietary factors: energy intake, fibre, red and processed meat, fruit, vegetables, sugary drinks.
ARIC, Atherosclerosis Risk in Communities; CARDIA, Coronary Artery Risk Development in Young Adults Study; ELSA-Brasil, Brazilian Longitudinal Study of Adult Health; MESA, Multi-Ethnic Study of Atherosclerosis; WHI, Women Health Initiative; FMC, Finnish Mobile Clinic Health Examination Survey; NOWAC, Norwegian Women and Cancer; SMC, Swedish Mammography Cohort; SUN, Seguimiento Universidad de Navarra (University of Navarra Follow-up); AusDiab, Australian Diabetes, Obesity and Lifestyle Study; CKB, China Kadoorie Biobank; JPHC, Japan Public Health Center-based; NHARC, Nutrition and Health of Aging Population of China Study; SWHS, Shanghai Women Health Survey

Table S1. Characteristics of the 28 contributing cohort studies in the InterConnect project on the association between fish intake and type 2 diabetes.

Study name	Country	Original inclusion/exclusion criteria	Recruitment time-frame	Baseline sample

Atherosclerosis Risk in Communities (ARIC)	US	Ethnically-representative men and women in 4 communities	1987-1989	15,792
Australian Diabetes, Obesity and Lifestyle Study (AusDiab)	Australia	All non-institutionalized adults >25 years of age residents in any of 42 randomly selected census collection districts in Australia	1999-2000	11,247
Brazilian Longitudinal Study of Adult Health (ELSA-Brasil)	Brazil	All active or retired employees of 6 institutions aged 35-74	2008-2010	15 105
China Kadoorie Biobank (CKB)	China	All adults aged 35–74 without major disability permanently resident and in each administrative unit (village or street committee) in 10 specific study areas (5 rural, 5 urban)	2004-2008	515,681
Cohort of Swedish Men (COSM) and Swedish Mammography Cohort (SMC)*	Sweden	All men born in 1918-1952 living in Västmanland and Örebro counties. All women living in Uppsala county born 1914-1948 and Västmanland County born 1917-1948	1987-1997	107,339
Coronary Artery Risk Development in Young Adults Study (CARDIA)	US	Individuals 18-30 years old socially and ethnically-stratified to be representatives of 4 cities	1985-1986	5,100
EPIC-InterAct (comprising 8 cohorts)	Denmark, France, Germany, Italy, the Netherlands, Spain, Sweden, UK	A Case-cohort of all type 2 diabetes cases occurring in European Prospective Investigation into Cancer and Nutrition (EPIC) cohorts between 1991 and 2007 from 8 of the 10 EPIC countries and a subcohort of individuals randomly selected from those with available stored blood and buffy coat, stratified by centre.	1991-1998	12,403 (cases); 16,154 (subcohort)
Finnish Mobile Clinic Health Examination Survey (FMC)	Finland	Men and women aged from 27 areas from different parts of Finland. The sample followed the age-distribution of the whole population..	1967-1972	10,054
Golestan study	Iran	Healthy 40-75 years old individuals (80% from rural areas and 75% of Turkmen ethnicity) living in Golestan	2004-2008	50,000

Hoorn study	The Netherlands	All men and women aged 60-75 years born between 1914 and 1940 living in the town of Hoorn	1989-1991	2,484
Japan Public Health Center-based (JPHC)	Japan	Registered residents aged 40-69 years within 11 public health center areas nationwide	1990-1994	113,000
Multi-Ethnic Study of Atherosclerosis (MESA)	US	Ethically-stratified asymptomatic men and women aged 45-84	2000-2002	6,814
Norwegian Women and Cancer (NOWAC)	Norway	All women have been sampled randomly from the Norwegian Central Person Register	1991-1997	102,540
Nutrition and Health of Aging Population of China (NHAPC) Study	China	Non-institutionalized individuals aged 50–70 from One rural county and two urban districts representing low, middle and upper socioeconomic levels in Beijing and Shanghai not diagnosed with cancer, coronary heart disease, stroke, Alzheimer's disease, dementia or any communicable disease	2005	3,533
Puerto Rico Heart Health Program (PRHHP)	Puerto Rico	Men aged 45-64 years representing the urban/rural composition of the island (approximately 70% urban and 30% rural)	1965-1968	8,793
Seguimiento Universidad de Navarra (SUN)	Spain	All university graduates from the University of Navarra, and university graduates from professional associations, willing to commit themselves for returning questionnaires every 2 years.	2000 - ongoing	4,717 (2000); 16,390 (2005)
Shanghai Men Health Study (SMHS)	China	Men aged 40-74 years in eight communities socially representatively of the general population of urban Shanghai.	2002-2006	61,480
Shanghai Women Health Study (SWHS)	China	Women aged 40-70 years in seven communities socially representatively of the general population of urban Shanghai.	1997-2000	75,000
Whitehall II	UK	all civil servants (men and women) aged 35–55 years working in the London offices of 20 Whitehall departments	1985-1988	10,308
Women Health Initiative (WHI)	US	Postmenopausal women	1994-1998	93,676

Zutphen Elderly	Netherlands	Men aged 65-84 who had participated in the Seven Countries Study and a similar sample of all other men of the same age group who were also living in Zutphen	1985	1,266

* For men we used data from COSM, and for women we used data from SCM.

Table S2. Dietary assessment methods and fish items available in the cohort studies participating in the InterConnect project on the association between fish intake and type 2 diabetes

Study	Description	Fish items
Atherosclerosis Risk in Communities (ARIC) USA	• Interviewer-administered semi-quantitative FFQ.<br>• 66 items<br>• Usual intake over the past year according to 9 frequency categories, ranging from never or <1 time/mo to ≥6 times/d.<br>• Standard portion sizes given as a reference for intake estimation.<br>• Supplementary questions included regarding frequency of fried food consumption and brand name of the breakfast cereal most commonly consumed (open-ended response).<br>Related References: Willett WC, et al. Am J Epidemiol. Jul 1985;122(1):51-65. (19)<br>Stevens J et al. Nutrition Research 1996;16: 735-745. (20)	4 items:<br>• Canned tuna fish;<br>• Dark meat fish;<br>• Other fish;<br>• Shrimp, lobster, scallops
Australian Diabetes, Obesity and Lifestyle Study (AusDiab)	• Semi-quantitative FFQ<br>• 101 items.<br>• Usual intake over the past year by checking 1 of 10 frequency categories ranging from 'never' to 'three or more times per day'.<br>• Recorded the amount and types of specific food items consumed by participants.<br>• In some cases pictures of serving sizes were provided so that persons could indicate whether they had more or less of a given food item each day and each week,<br>Related References: Grantham N.M., Magliano D.J., Hodge A., Jowett J., Meikle P., Shaw J.E. The association between dairy food intake and the incidence of diabetes in Australia: The Australian diabetes obesity and lifestyle study (AusDiab) Public Health Nutr.2013;16:339–345.	3 items:<br>• Fish - steamed, grilled or baked<br>• Fried fish including take away<br>• Fish - tinned (salmon, tuna, sardines etc.)
Brazilian Longitudinal Study of Adult Health (ELSA-Brasil, Brazil)	• Semi-quantitative FFQ filled out online during the participants' interviews<br>• 114 items<br>• Usual consumption with 8 frequency options varying from "More than 3x daily" to "Never/Almost never"<br>• Portion sizes given<br>• In order to include seasonal consumption, one more column was included where it is possible to record the occurrence of spontaneous manifestation, such as "only during a certain period", "only during the season", or any other expression that indicates regular consumption during a certain time of the year.	6 items:<br>• Roasted/cooked fish (g/day)<br>• Fried fish (g/day)<br>• Canned sardines/tuna (g/day)<br>• Shrimp/mussels (g/day)<br>• Crab (g/day)<br>• Fish stew (g/day)

Table S2. Dietary assessment methods and fish items available in the cohort studies participating in the InterConnect project on the association between fish intake and type 2 diabetes

Study	Description	Fish items
	• The Food Frequency Questionnaire used in ELSA Brasil uses the Nutrition Data System for Research (NDSR) table and *Tabela Brasileira de Composição de Alimentos* (TACO) (http://www.unicamp.br/nepa/taco/) for calculating the dietary nutrients.<br>Related References: Molina MCB et al. Diet assessment in the Brazilian Longitudinal Study of Adult Health (ELSA-*Brasil*): Development of a food frequency questionnaire. Rev. Nutr. vol.26 no.2 Campinas Mar./Apr. 2013	
China Kadoorie Biobank (CKB, China)	• Basic FFQ. Trained health workers administered the FFQ as a laptop-based questionnaire<br>• 12 major food groups in China: rice, wheat, other staples (such as corn, millet, etc.), meat, poultry, fish, eggs, fresh fruit, fresh vegetables, preserved vegetables, soybean, and dairy products.<br>• Dietary habits during the preceding 12 months using five frequency levels of habitual consumption: never/rarely, monthly, 1–3 days/week, 4–6 days/week, or daily).<br>Related References: Yu C, Shi Z, Lv J, Guo Y, Bian Z, Du H, Chen Y, Tao R, Huang Y, Chen J, Chen Z, Li L.Dietary Patterns and Insomnia Symptoms in Chinese Adults: The China Kadoorie Biobank Nutrients. 2017 Mar 4;9(3)	1 item: total fish
Cohort of Swedish Men (COSM) and Swedish Mammography Cohort (SMC)	• Validated FFQ.<br>• 96-item<br>• usual consumption during the past year, filling in 1 of 8 predefined frequency: 0 servings per month, 1-3 servings per month, 1-2 servings per week, 3-4 servings per week, 5-6 servings per week, 1 serving per day, 2 servings per day, ≥3 servings per day)<br>Related References: Wolk A, Larsson SC, Johansson J-E, Ekman P. Long-term fatty fish consumption and renal cell carcinoma incidence in women. JAMA 2006 Sep;296(11):1371-76.<br>Thomas LD, Michaelsson K, Julin B, Wolk A, Akesson A. Dietary cadmium exposure and fracture incidence among men: a population-based prospective cohort study. J Bone Miner Res2011;26:1601-8	5 items:<br>• Herring/mackerel<br>• Salmon/whitefish/ red char<br>• Cod/saithe/fish sticks<br>• Caviar<br>• Shellfish (shrimp/crayfish)

Table S2. Dietary assessment methods and fish items available in the cohort studies participating in the InterConnect project on the association between fish intake and type 2 diabetes

Study	Description	Fish items
Coronary Artery Risk Development in Young Adults (CARDIA)	• Interviewer-administered, validated diet history.<br>• 100 food categories; 166 food groups using the food-grouping system devised by the Nutrition Coordinating Center at the University of Minnesota.<br>• Usual consumption in the previous month within to which a positive response would trigger the posing of additional information-gathering questions. For example, a positive response to the question "Do you eat any fast food hamburgers or cheeseburgers?" would trigger the additional questions "What kind do you have?" "How many?" "How often?"<br>• Recipes were decomposed into constituent foods, assigned a number of servings per day, and added to the appropriate food group.<br>Related References: Shikany JM, Jacobs DR Jr, Lewis CE, Steffen LM, Sternfeld B, Carnethon MR, Richman JS. Associations between food groups, dietary patterns, and cardiorespiratory fitness in the Coronary Artery Risk Development in Young Adults study. Am J Clin Nutr. 2013 Dec;98(6):1402-9	5 items:<br>• Fish – fresh and cooked<br>• Lean fish – fresh and cooked<br>• Fried fish – commercial entrée and fast food<br>• Shellfish<br>• Fried shellfish - commercial entrée and fast food
EPIC-InterAct (comprising 8 cohorts)	• Quantitative dietary questionnaire with individual average portion sizes (in France, Spain, Netherlands, Germany, and Italy, except Naples) or a semiquantitative food-frequency questionnaire [in Denmark, Naples (Italy), Sweden, and the United Kingdom], which were developed and validated locally.<br>• Dietary intake data from each EPIC cohort were standardized by using the EPIC Nutrient Database, which was developed to provide standardized information on food pattern, food items, and individual nutrients across EPIC countries<br>Related References: Patel PS, Forouhi NG, Kuijsten A, Schulze MB, van Woudenbergh GJ, Ardanaz E, Amiano P, Arriola L, Balkau B, Barricarte A, Beulens JW, Boeing H, Buijsse B, Crowe FL, de Lauzon-Guillan B, Fagherazzi G, Franks PW, Gonzalez C, Grioni S, Halkjaer J, Huerta JM, Key TJ, Kühn T, Masala G, Nilsson P, Overvad K, Panico S, Quirós JR, Rolandsson O, Sacerdote C, Sánchez MJ, Schmidt EB,	4 items:<br>• crumbs and nonspecific or combined fish)

Table S2. Dietary assessment methods and fish items available in the cohort studies participating in the InterConnect project on the association between fish intake and type 2 diabetes

Study	Description	Fish items
	Slimani N, Spijkerman AM, Teucher B, Tjonneland A, Tormo MJ, Tumino R, van der A DL, van der Schouw YT, Sharp SJ, Langenberg C, Feskens EJ, Riboli E, Wareham NJ; InterAct Consortium. The prospective association between total and type of fish intake and type 2 diabetes in 8 European countries: EPIC-InterAct Study. Am J Clin Nutr. 2012 Jun;95(6):1445-53.	
Finnish Mobile Clinic Health Examination Survey (FMC)	• Dietary history interview<br>• Habitual food consumption over the preceding year.<br>• The interview was guided by a preformed questionnaire and artificial food models and pieces of real food were available to assist in estimating the amounts of food.<br>• Generally, the time taken for the interview was 30 to 40 minutes however the individual variation was great.<br>• The intakes of different food items and nutrients were calculated based on the interview data. The nutrient composition database used initially was founded on the contemporary Finnish food composition tables. During late eighties, the nutrient composition database was updated based on the food composition tables published by the Finnish Social Insurance Institution.<br>Related References: Knekt P, Rissanen H, Järvinen R, Heliövaara M. Cohort Profile: The Finnish Mobile Clinic Health Surveys FMC, FMCF and MFS. Int J Epidemiol. 2017 Dec 1;46(6):1760-1761i. doi: 10.1093/ije/dyx092	24 fish items:<br>• Perch<br>• Pike<br>• Flounder<br>• Bream<br>• Salmon<br>• Vendace, with bones<br>• Fresh, frozen, saithe<br>• Whitefish<br>• Baltic herring, with bones<br>• Baltic herring<br>• Cod<br>• Fish, average<br>• Fish in soup, average<br>• Shrimp<br>• Roe<br>• Stockfish<br>• Vendace, Salted with bones<br>• Salmon, slated<br>• Baltic herring, slated with bones<br>• Herring, salted<br>• Smoked vendace with bones<br>• Smoked lamprey<br>• Smoked redfish<br>• Smoked whitefish<br>• Smoked Baltic herring with bones<br>• Smoked fish, average<br>• Sardine<br>• Tuna

Table S2. Dietary assessment methods and fish items available in the cohort studies participating in the InterConnect project on the association between fish intake and type 2 diabetes

Study	Description	Fish items
Golestan Study	• Semi-quantitative FFQ<br>• 150 items<br>• Usual food intake over the previous year.<br>• Frequency of food intake was recorded in times per day, week, month and year or as never.<br>• Portion size was estimated as the median of each item. Pictures of different portion sizes were used for 51 food items to increase the precisions of the estimates<br>Related References: Malekshah AF, Kimiagar M, Saadatian-Elahi M, et al. Validity and reliability of a new food frequency questionnaire compared to 24 h recalls and biochemical measurements: pilot phase of Golestan cohort study of esophageal cancer. Eur J Clin Nutr. 2006;60(8):971–977.	5 items:<br>• Sturgeon<br>• Carp<br>• River fish<br>• Tuna<br>• Smoked fish<br>• Salted fish<br>5 cooking methods:<br>• Shallow fry<br>• Deep fry<br>• Bbq<br>• Boil<br>• Steam
Multi-Ethnic Study of Atherosclerosis (MESA)	• Self-administered, modified-Block FFQ<br>• 120 items<br>Related References: Mayer-Davis E et al. Ann Epidemiol 1999;9:314–324. & Nettleton JA et al. Br J Nutr 2009; 102, 1220–1227	6 items:<br>• Shrimp, lobster, crab, oysters, mussels (not fried);<br>• Tuna, salmon, sardines (including sashimi or sushi);<br>• Other broiled, steamed, baked or raw fish (trout, sole, halibut, poke, grouper);<br>• Fried fish or fish sandwich, fried shrimp, calamari;<br>• Fish stew or seafood gumbo, paella;<br>• Stir-fried shrimp or fish with vegetables;

Table S2. Dietary assessment methods and fish items available in the cohort studies participating in the InterConnect project on the association between fish intake and type 2 diabetes

Study	Description	Fish items
Hoorn study	• Validated semi-quantitative FFQ<br>• 92-items.<br>• The participants filled in the questionnaire at home, and the FFQs were checked in the research center for completeness<br>Related References: Grootenhuis PA, Westenbrink S, Sie CMTL, De Neeling N, Kok FJ and Bouter LM. A Semiquantitative food frequency questionnaire for use in epdimeiologic research among the elderly: validation by comparison with dietary history. J Clin Epi, 1995.	24 Items:<br>• 10 items of fish with bread as a 20g serving (herring, mackerel, shrimp/tuna, mussels, tuna, herring in tomato sauce, fried codfish, sardines, canned salmon, eel, other fish)<br>• 6 items of fish as a main hot lunch meal as a 100g serving (codfish, tuna/salmon, herring, mackerel, tuna/salmon, shrimps/mussels, other fish)<br>• 8 items of fish as a main hot dinner meal as a 100g serving (sprat, plaice, shrimps/mussels, herring, mackerel, sardines, eel, fried codfish, other fish
Japan Public Health Center–based Prospective (JPHC) Study	• Validated FFQ<br>• 147 food and beverage items<br>• Usual consumption over the previous year.<br>• For most food items, 9 response options were available to describe consumption frequency, ranging from rarely (<1 time/mo) to ≥7 times/d. A standard portion size was specified for each food, and respondents were asked to denote their usual portion size from 3 options (≥0.5 times, standard, or ≥1.5 times).<br>• The daily intake of fish/seafood was calculated by multiplying daily consumption frequency by the typical portion size.<br>Related References: Nanri A., Mizoue T., Noda M., Takahashi Y., Poudel T., Kato M., Oba S., Inoue M., Tsugane S. Fish intake and type 2 diabetes in Japanese men and women: The Japan Public Health	19 items:<br>• 7 fresh fish: salmon, skipjack/tuna, cod/flatfish, sea bream, horse mackerel/sardine, saury/mackerel, and eel;<br>• 5 seafood other than fish: squid, octopus, shrimp, clam, and pond snail;<br>• 4 salted and dried fish products: salted fish, dried fish, dried whitebait, and salted fish roe;

Table S2. Dietary assessment methods and fish items available in the cohort studies participating in the InterConnect project on the association between fish intake and type 2 diabetes

Study	Description	Fish items
	Center-based Prospective Study. Am. J. Clin. Nutr. 2011;94:884–891	• 1 other fish product: canned tuna;<br>• 2 items of fish-paste products (*chikuwa* and *kamaboko*)
Norwegian Women and Cancer study (NOWAC)	• FFQ<br>• 132 items<br>• Habitual diet over the past year, with emphasis on fish consumption and a traditional diet in the study population.<br>• Similar items were grouped together in blocks with question headings. The response options were predefined and listed in increasing order with check-boxes to facilitate completion and optical reading. For example, the items listed under the question "How often do you eat fruit?" were "apples/pears", "oranges", "bananas", and "other fruit" with the following options: "never/rarely", "1–3 per month", "1 per week", "2–4 per week", "5–6 per week", "1 per day", and "2+ per day". The first alternative for consumption frequencies was always "never/rarely", but the number of options ranged from 4 to 7 depending on the food. When convenient, the questions were phrased in terms of natural units, such as glasses (milk, fruit juice, soft drinks, and wine), cups (coffee), slices (bread), or number (eggs and potatoes).<br>• Separate questions about the usual amounts consumed were included for fat on bread, vegetables, fish and fish products, sauces and condiments for fish, meat and meat products, ice cream, chocolate, and cod liver oil supplements. The number of response options ranged from 3 to 5 with units in pieces, slices, decilitres, florets (broccoli and cauliflower), or spoonfuls.<br>Related References:<br>CL Parr, MB Veierod , P Laake , E Lund & A Hjartaker (2006) Test–retest reproducibility of a food frequency questionnaire (FFQ) and estimated effects	8 items:<br>• Includes boiled or fried cod, saithe, haddock, pollack<br>• Includes catfish, salmon, herring, mackerel, flounder, trout, redfish<br>• Includes other kind of fish not listed above<br>• Includes fish cakes, battered fish, fish au gratin<br>• Includes shrimps,<br>• Includes fish row, mainly from cod<br>• Includes fish liver, mainly from cod<br>• Includes spread for bread containing, mackerel, caviar, salmon, herring and other kind of fish spread

Table S2. Dietary assessment methods and fish items available in the cohort studies participating in the InterConnect project on the association between fish intake and type 2 diabetes

Study	Description	Fish items
	on disease risk in the Norwegian Women and Cancer Study (NOWAC). *Nutr J* 5, 4.	
Nutrition and Health of Aging Population of China (NHAPC) Study	• Validated FFQ used in the 2002 National Nutrition and Health Survey in China<br>• 74 items<br>• Nutrients were estimated according to the Chin ese Food Composition Tables .<br>Related reference： Yang YX, Wang GY, Pan XC (Eds.). China Food Composition Tables 2002. Beijing, China, Beijing University Medical Press, 2002<br>Zhao W, Hasegawa K, Chen J. The use of food-frequency questionnaires for various purposes in China. Public Health Nutr 2002; 5:829–833	7 items:<br>• Freshwater fish (Goldfish carp, Fatheaded carp, Common carp, Grass carp/Black snail carp, Japanese sea bass, etc)<br>• Saltwater fish (Belt fish, Yellow croaker, Flatfish, Butterfish, etc)<br>• Shrimp and Crab (Shrimp, freshwater, Shrimp, marine, Crab, freshwater, Sea crab)<br>• Shellfish (Scallop, Venus clam, White clam, Clam, Rice-paddy field snail, Oyster, etc.)<br>• Soft - bodied fisheries products (Jellyfish body, Squid, Sea cucumber, Cuttlefish, etc)<br>• Hairy shrimps/ Dried shrimps<br>• Salted fish
Puerto Rico Heart Health Program (PRHHP)	• 24-h recall<br>• Food models and standard-sized utensils were used to obtain a quantitative assessment from participants.<br>• Intake of energy and macronutrients was calculated using the United States Department of Agriculture Handbook #8 food composition tables or other more direct sources of nutrients analysis for foods special to Puerto Rico.<br>Related references: Smit E, Garcia-Palmieri MR, Figueroa NR, McGee DL, Messina M, Freudenheim JL, Crespo CJ.Protein and legume	3 items:<br>• Fresh fish<br>• Other seafood and shellfish<br>• Salt cod

Table S2. Dietary assessment methods and fish items available in the cohort studies participating in the InterConnect project on the association between fish intake and type 2 diabetes

Study	Description	Fish items
	intake and prostate cancer mortality in puerto rican men. *Nutr Cancer*. 2007;58:146–52	
Seguimiento Universidad de Navarra (SUN)	• Semi quantitative FFQ validated and repeatedly re-evaluated in Spain<br>• 136-items; frequencies of consumption in nine categories (from never to more than 6 servings a day)<br>• Food frequency questions included a typical portion size for each item<br>Martín-Moreno JM, Boyle P, Gorgojo L, et al. Development and validation of a food frequency questionnaire in Spain. Int J Epidemiol 1993;22:512-9. doi:10.1093/ije/22.3.512	
Shanghai Men's Health Study (SMHS) and Shanghai Women's Health Study (SWHS)	• Validated FFQ that covers about 90% of commonly consumed foods in urban Shanghai in 1996.<br>• 77 items in SWHS and 81 in the SMHS<br>• For each food item or food group, subjects were asked how frequently (daily, weekly, monthly, yearly, or never) they consumed the food or food groups, followed by a question of amount of consumption in lians (50 g/lian) per unit of time.<br>• For seasonal foods (mainly vegetables and fruits), in-season consumption pattern was elicited.<br>Related references: Shu XO, Yang G, Jin F, et al. Validity and reproducibility of the food frequency questionnaire used in the Shanghai Women's Health Study. Eur J Clin Nutr 2004;58:17–23<br>Cai H, Zheng W, Xiang YB, et al. Dietary patterns and their correlates among middle-aged and elderly Chinese men: a report from the Shanghai Men's Health Study. Br J Nutr 2007;98:1006–	6 items:<br>• Salt water fish (e.g., yellow croaker, hair tail)<br>• Fresh water fish (e.g., silver carp, bream, crucian, cart, ect.)<br>• Rice-field eel or river eel<br>• Eat salted fish<br>• Shrimp, crab, ect<br>• Conch, etc.
Whitehall II	• Validated item food frequency questionnaire<br>• 127 items.<br>• For each of the food items, participants self-reported how often on average they had consumed a common unit or portion size in 1 of 9 categories during the previous year.<br>Related references: Ozawa M, Shipley M, Kivimaki M, et al. Dietary pattern, inflammation and	5 items:<br>• Fried fish in batter as in fish and chips<br>• Fish fingers of Fish cakes<br>• Other white fish, fresh or frozen

Table S2. Dietary assessment methods and fish items available in the cohort studies participating in the InterConnect project on the association between fish intake and type 2 diabetes

Study	Description	Fish items
	cognitive decline: The Whitehall II prospective cohort study. Clin Nutr. 2017;36:506–12.	eg cod, haddock, plaice, sole, halibut<br>• Oily fish, fresh or canned eg mackerel, kippers, tuna, salmon, sardines, herring<br>• Shellfish eg crab, prawns, mussels
Women Health Initiative (WHI)	• Standardised self-administered FFQ<br>• 122 items<br>• Average daily nutrient intake over the previous 3-month period.<br>• The nutrient database, linked to the University of Minnesota Nutrition Data System for Research (NDSR) is based on the US Department of Agriculture Standard<br>Reference Releases and manufacturer information. Related references: Patterson RE, Kristal AR, Tinker LF, Carter RA, Bolton MP, Agurs-Collins T. Measurement characteristics of the Women's Health Initiative food frequency questionnaire. Ann Epidemiol.1999;9:178–187.	5 items:<br>• fried fish, fish sandwich, fried shellfish (3 ounces/1 sandwich)<br>• shellfish, not fried (shrimp, lobster, crab, and oysters) (3 ounces or ½ cup),<br>• canned tuna, tuna salad, and tuna casserole (1/2 cup tuna or 1 cup casserole)<br>• white fish (broiled or baked) (sole, snapper, cod) (3 ounces)<br>• dark fish (broiled or baked) (salmon, mackerel, bluefish) (3 ounces).
Zutphen Elderly Study	• Cross-check dietary history method adapted to the Dutch setting recorded by trained dietitians<br>• Usual food intake of the participants in the month before the interview<br>• Each participant was interviewed at home for about 1 h together with the person who prepared the food (in most cases the wife) about his usual food consumption pattern on weekdays and at weekends, and about food purchases.<br>• The habitual consumption of foods during a week was assessed and verified with the	57 items:<br>• 14 canned fish items<br>• 10 fatty fish items (fat>12-15g)<br>• 21 lean fish items<br>• 12 shellfish items

Table S2. Dietary assessment methods and fish items available in the cohort studies participating in the InterConnect project on the association between fish intake and type 2 diabetes

Study	Description	Fish items
	quantities of foods bought per week. This information was combined to calculate the participant's food consumption on a typical weekday.<br>• Quantities of foods were estimated by the dietitians, with a portable scale if necessary.<br>• The food intake data were encoded by the dietitians according to the Netherlands Uniform Food Encoding System, and converted into energy and nutrient values by the 1985 release of the Netherlands nutrient data bank updated with 1993 data for beta-carotene, vitamin E, and with flavonoid data.<br>Related references: Hertog M. G., Feskens E. J., Hollman P. C., Katan M. B., Kromhout D. Dietary antioxidant flavonoids and risk of coronary heart disease: the Zutphen elderly study. *The Lancet*.1993;342(8878):1007–1011	

Table S3. Assessment of confounders in the cohort studies participating in the InterConnect project on the association between fish intake and type 2 diabetes.

Study	Description
Atherosclerosis Risk in Communities (ARIC)	Education: 1=Education Level = Basic Education or 0 Years Education; 2=Education Level = Intermediate Education; 3=Education Level = Advanced Education<br>Smoking: Cigarette Smoking Status (1=Current smoker, 2=Former smoker, 3=Never smoker. 4=unknown but one of the above three categories may be ruled out.<br>Physical activity: Sport during leisure time (continuous, values range: 1-5)<br>Alcohol intake: Ethanol Intake in g/wk<br>BMI and Waist circumference: measured<br>Family history of diabetes: Y/N (if mother or dad)<br>Co-morbidity available: heart attack, stroke, hypertension assessed by questionnaire<br>Total energy intake; Fibre; Red and processed meat; Fruits; Vegetables; Sugary beverages, Fish-oil supplement (NA): FFQ
Australian Diabetes, Obesity and Lifestyle Study (AusDiab)	Education: 1 university, 2 completed high school, 3 some high school, 4 primary school or never attended school<br>Smoking: 3= non smoker, 2= ex smoker, 1= Current smoker<br>Physical activity: 1= sedentary (no PA), 2= Insufficient (b/w 0 and 150 mins, phys. act. time), 3=Sufficient (over 150 mins phys. act. time)<br>Alcohol intake: Alcohol (g/day)<br>BMI and Waist circumference: measured<br>Family history of diabetes: questionnaire<br>Co-morbidity available: heart attack, stroke, hypertension assessed by questionnaire<br>Total energy intake; Fibre; Red and processed meat; Fruits; Vegetables; Fish-oil supplement (NA): FFQ

Table S3. Assessment of confounders in the cohort studies participating in the InterConnect project on the association between fish intake and type 2 diabetes.

Study	Description
Brazilian Longitudinal Study of Adult Health (ELSA-Brasil)	Education: 1= incomplete elementary school; 2= complete elementary school; 3= complete secondary school; 4= university degree; missing = -1<br>Smoking: 0 = never smoked; 1 = former smoker; 2 = smoker; missing = -1<br>Physical activity: Adjusted sum of minutes of leisure time physical activity (minutes of MET/week)<br>Alcohol intake: Quantity of alcohol intake/week (grams)<br>BMI and Waist circumference: measured<br>Family history of diabetes: Diabetes in the family<br>Co-morbidity available: Myocardial infarction, stroke, cancer, hypertension assessed by questionnaire<br>Total energy intake; Fibre; Red and processed meat; Fruits; Vegetables; Sugary beverages, Fish-oil supplement: (NA) FFQ
China Kadoorie Biobank (CKB)	Education: 0= no formal school; 1= primary school; 2= middle school; 3= high school; 4= technical school/college; 5= university<br>Smoking: 1= never smoker; 2= occasional smoker; 3=ex regular smoker; 4=smoker<br>Physical activity: Total physical activity (MET-hr/day)<br>Alcohol intake: Average grams of alcohol consumed in a typical week)<br>BMI and Waist circumference: measured<br>Family history of diabetes: Father, mother, brother, sister with diabetes<br>Co-morbidity available: cardiovascular disease, stroke, cancer, hypertension assessed by questionnaire<br>Total energy intake (NA); Fibre (NA); Red and processed meat; Fruits; Vegetables; Sugary beverages (NA), Fish-oil supplement: FFQ

Table S3. Assessment of confounders in the cohort studies participating in the InterConnect project on the association between fish intake and type 2 diabetes.

Study	Description
Cohort of Swedish Men (COSM) and Swedish Mammography Cohort (SMC)	Education: 1=Up to 9 years, 2=10-12 years, 3=more than 12 year<br>Smoking: 1=Current, 2=Ex, 3=Never<br>Physical activity: Current total activity score (MET*hours/d)<br>days/w 5=Almost daily<br>Alcohol intake: Alcohol (g) calculated from FFQ<br>BMI and Waist circumference: measured<br>Family history of diabetes: 1=Yes 0=No (Father, Mother, Sibling)<br>Co-morbidity available: heart attack, stroke, cancer, high blood pressure assessed by questionnaire and patient registries<br>Total energy intake; Fibre; Red and processed meat; Fruits; Vegetables; Sugary beverages (Fruit juice(saft)/soda), Fish-oil supplement: FFQ
Coronary Artery Risk Development in Young Adults (CARDIA)	Education: 1 university, 2 completed high school, 3 some high school, 4 primary school or never attended school<br>Smoking: 3= non smoker, 2= ex smoker, 1= Current smoker<br>Physical activity: 1= sedentary (no PA), 2= Insufficient (b/w 0 and 150 mins, phys. act. time), 3=Sufficient (over 150 mins phys. act. time)<br>Alcohol intake: Alcohol (g/day)<br>BMI and Waist circumference: measured<br>Family history of diabetes: questionnaire<br>Co-morbidity available: heart attack, stroke, hypertension assessed by questionnaire<br>Total energy intake; Fibre; Red and processed meat; Fruits; Vegetables; Sugary beverages, Fish-oil supplement (NA): FFQ

Table S3. Assessment of confounders in the cohort studies participating in the InterConnect project on the association between fish intake and type 2 diabetes.

Study	Description
EPIC-InterAct (8 cohorts)	Education: 0 = None; 1 = Primary school completed; 2 = Technical/professional school; 3 = Secondary school; 4 = Longer education (incl. University deg.); 5 = Not specifiedSmoking: (never; formerl; smoker; unknown)<br>Physical activity: pa_index (1=inactive, 2=moderately inactive, 3=moderately active, 4=active<br>Alcohol intake: Alcohol (g/day)<br>BMI and Waist circumference: measured<br>Family history of diabetes: (in parent or sibling)<br>Co-morbidity available: heart attack, stroke, hypertension assessed by questionnaire<br>Total energy intake; Fibre; Red and processed meat; Fruits; Vegetables; Sugary beverages, Fish-oil supplement (NA): FFQ
Finnish Mobile Clinic Health Examination Survey (FMC)	Education: NA<br>Smoking: 1 = HAS NEVER SMOKED; 2 = EX-SMOKER; 3= ONLY PIPE OR CIGAR; 4 = LESS THAN 15 CIGARETTES A DAY; 5 = MORE THAN 15 CIGARETTES A DAY<br>Physical activity: How often exercise during leisure time:1=Almost never 2=1-3 days/m 3=1-2 days/w 4=3-4 days/w 5=Almost daily<br>Alcohol intake: NA<br>BMI and Waist circumference: measured<br>Family history of diabetes: 1=Yes 0=No (Any relative)<br>Co-morbidity available: heart disease, cancer, hypertension assessed by questionnaire<br>Total energy intake; Fibre; Red and processed meat; Fruits; Vegetables; Fish-oil supplement (NA): FFQ
Golestan Study	Education: 1= illiterate, 2=formal education≤5 years, 3=6-8years, 4=9-12 years, 5=university<br>Smoking: 0=never 1=current 2=former<br>Physical activity: 1=irregular non-intense, 2= regular non-intense, 3=irregular or regular intense,<br>Alcohol intake: 0=never 1=ever<br>BMI and Waist circumference: measured<br>Family history of diabetes: NA<br>Co-morbidity available: Heart disease, stroke, cancer, hypertension assessed by questionnaire

Table S3. Assessment of confounders in the cohort studies participating in the InterConnect project on the association between fish intake and type 2 diabetes.

Study	Description
	Total energy intake; Fibre; Red and processed meat; Fruits; Vegetables; Sugary beverages, Fish-oil supplement (NA): FFQ
Multi-Ethnic Study of Atherosclerosis (MESA)	Education: 0=NO SCHOOLING 1=GRADES 1-8 2=GRADES 9-11 3=COMPLETED HIGH SCHOOL/GED 4=SOME COLLEGE BUT NO DEGREE 5=TECHNICAL SCHOOL CERTIFICATE 6=ASSOCIATE DEGREE 7=BACHELOR'S DEGREE 8=GRADUATE, PROFESSIONAL SCHOOL<br>Smoking: 0= never, 1= former, 1= Current<br>Physical activity: Total leisure MET-min/wk<br>Alcohol intake: Alcohol (g/day)<br>BMI and Waist circumference: measured<br>Family history of diabetes: NA<br>Co-morbidity available: cancer, hypertension assessed by questionnaire<br>Total energy intake; Fibre; Red and processed meat; Fruits; Vegetables; Sugary beverages, Fish-oil supplement (Cod liver oil, other fish oils or omega-3 fatty acids): FFQ
Hoorn study	Education: 1= primary education; 2=low vocational education; 3= intermediate secondary education; 4= intermediate vocational education; 5=high secondary education; 6= high vocational education; 7=BA or university; 8= different<br>Smoking: 0=non-smoker cigarettes; 1=smoker cigarettes; 2=former smoker cigarettes<br>Physical activity: Numerical score (0 to 8) derived from 9 equally weighted y/n questions about regularly performing the following activities: walking, bicycling, playing sports, doing odd jobs, gardening, doing household activities, climbing stairs, doing shopping, and working<br>Alcohol intake: g/d<br>BMI and Waist circumference: measured<br>Family history of diabetes: 1=Yes 0=No (father, Mother, Brother, Sister)

Table S3. Assessment of confounders in the cohort studies participating in the InterConnect project on the association between fish intake and type 2 diabetes.

Study	Description
	Co-morbidity available: f cardiovascular disease, cancer, hypertension assessed by questionnaire<br>Total energy intake; Fibre; Red and processed meat; Fruits; Vegetables; Sugary beverages, Fish-oil supplement (NA): FFQ
Japan Public Health Center–based Prospective (JPHC) Study	Education: NA<br>Smoking: 0=never smoking, 1=past smoking, 2=current smoking<br>Physical activity: Frequency of any sports or exercises apart from work:1=Almost never 2=1-3 days/m 3=1-2 days/w 4=3-4 days/w 5=Almost daily<br>Alcohol intake: Frequency of drinking: 0=Almost never 1=1-3 days/month 2=1-2 days/week 3=3-4 days/week 4=5-6 days/week 5=Daily<br>BMI and Waist circumference: measured<br>Family history of diabetes: 1=Yes 0=No<br>Co-morbidity available: ischemic heart disease, stroke, cancer, hypertension assessed by questionnaire<br>Total energy intake; Fibre; Red and processed meat; Fruits; Vegetables; Sugary beverages, Fish-oil supplement (NA): FFQ
Norwegian Women and Cancer study (NOWAC)	Education: Total years in school<br>Smoking1=never, 2=former, 3=current<br>Physical activity: Please indicate on a scale from 1-10 how active you are. 1= very little, 10= very much<br>Alcohol intake: g/d<br>BMI and Waist circumference: measured<br>Family history of diabetes: 1=Yes 0=No<br>Co-morbidity available: MI, stroke, cancer, hypertension assessed by questionnaire

Table S3. Assessment of confounders in the cohort studies participating in the InterConnect project on the association between fish intake and type 2 diabetes.

Study	Description
	Total energy intake; Fibre; Red and processed meat; Fruits; Vegetables; Sugary beverages (lemonade, soda and juice), Fish-oil supplement (fish liver oil): FFQ
Nutrition and Health of Aging Population of China (NHAPC) Study	Education: Years of education: 1= 0-6 y, 2= 7-9 y, 3= 10~ y<br>Smoking: 1=current smoker; 2=no<br>Physical activity: Level: 1=low 2=median 3=heavy<br>Alcohol intake: 1=current drinker; 2=no<br>BMI and Waist circumference: measured<br>Family history of diabetes: 1=Yes 0=No<br>Co-morbidity available: coronary heart disease and MI, stroke, cancer, hypertension assessed by questionnaire<br>Total energy intake; Fibre; Red and processed meat; Fruits; Vegetables; Sugary beverages (lemonade, soda and juice, Fish-oil supplement: FFQ
Puerto Rico Heart Health Program (PRHHP)	Education: 0 none; 1 grades 1- 4 2 grades 5-8 3 high school - attended 4 high school - graduated 5 university - attended 6 university – graduated<br>Smoking: Smoking cigarettes: 0 newer smoked cigarettes; 1 smokes cigarettes now; 2 smoked cigarettes previously but not now<br>Physical activity: n of hours/d of mod activity<br>Alcohol intake: Alcohol (g/day)<br>BMI: measured<br>Family history of diabetes: Y/N (mother, father, siblings)<br>Co-morbidity available: cardiovascular disease, stroke, hypertension assessed by questionnaire<br>Total energy intake; Fibre; Red and processed meat; Fruits; Vegetables; Sugary beverages, Fish-oil supplement (NA): FFQ

Table S3. Assessment of confounders in the cohort studies participating in the InterConnect project on the association between fish intake and type 2 diabetes.

Study	Description
Seguimiento Universidad Navarra (SUN)	Education: None; doctoral thesis; degree; associate's degree; master<br>Smoking: 1=never, 2=former, 3=current<br>Physical activity: MET-hours/week<br>Alcohol intake: g/day<br>BMI and Waist circumference: measured<br>Family history of diabetes: 1=Yes 0=No<br>Co-morbidity available: coronary heart disease and MI, stroke, cancer, hypertension assessed by questionnaire<br>Total energy intake; Fibre; Red and processed meat; Fruits; Vegetables; Sugary beverages; Fish-oil supplement: FFQ
Shanghai Men's Health Study (SMHS) and Shanghai Women's Health Study (SWHS)	Education: No formal education; Elementary school; Middle school; high school; technical school/college or above<br>Smoking1=never, 2=former, 3=current<br>Physical activity: MET-h/day/yr<br>Alcohol intake: drinks/d<br>BMI and Waist circumference: measured<br>Family history of diabetes: 1=Yes 0=No<br>Co-morbidity available: Coronary heart disease, stroke, cancer, hypertension assessed by questionnaire<br>Total energy intake; Fibre; Red and processed meat; Fruits; Vegetables; Sugary beverages (lemonade, soda and juice), Fish-oil supplement: FFQ
Whitehall II	Education: number of years of education and highest level (5 cat)<br>Smoking: current smoker: Y/N<br>Physical activity: 0: low ; 1: moderate; 2 : intense (derived from 20 questionnaire items on frequency and duration)<br>Alcohol intake: glasses per day (estimated from FFQ)<br>BMI and Waist circumference: measured<br>Family history of diabetes: 1=Yes 0=No

Table S3. Assessment of confounders in the cohort studies participating in the InterConnect project on the association between fish intake and type 2 diabetes.

Study	Description
	Co-morbidity available: ischemic heart disease, stroke, cancer, hypertension assessed by questionnaire<br>Total energy intake; Fibre; Red and processed meat; Fruits; Vegetables; Sugary beverages, Fish-oil supplement: FFQ
Women Health Initiative (WHI)	Education: 11 categories, 1 lowest level<br>Smoking: 0= never smoker, 1= Past smoker, 2= Current smoker<br>Physical activity: MET-hours/week of recreational activity<br>Alcohol intake: Alcohol (g/day)<br>BMI and Waist circumference: measured<br>Family history of diabetes: Y/N (relative)<br>Co-morbidity available: cardiac arrest or angina, cancer, hypertension assessed by questionnaire<br>Total energy intake; Fibre; Red and processed meat; Fruits; Vegetables; Sugary beverages, Fish-oil supplement (NA): FFQ
Zutphen Elderly Study	Education: 1=Primary education; 2=Low level vocational training (Dutch: lbo). 3=Extended low level vocational training; 4=Vocational training (Dutch: mbo); 5=Secondary education (Dutch: hbs, lyceum or gymnasium); 6=High level applied training (Dutch: hts, mo-opleiding); 7=Higher education (university or college (Dutch: universiteit or hogeschool); 8=Other<br>Smoking: 0=never smoking, 1=former smoking, 2=current smoking<br>Physical activity: 1=Not active at intensity >4 MET; 2= 1-150 minutes active at intensity >4 MET; 3= >150 minutes active at intensity >4 MET<br>Alcohol intake: g/d<br>BMI: measured<br>Family history of diabetes: 1=Yes 0=No<br>Co-morbidity available: MI, stroke, hypertension assessed by questionnaire

Table S3. Assessment of confounders in the cohort studies participating in the InterConnect project on the association between fish intake and type 2 diabetes.

Study	Description
	Total energy intake; Fibre; Red and processed meat; Fruits; Vegetables; Sugary beverages, Fish-oil supplement (NA): FFQ

Table S4. Incidence Rate Ratio of type 2 diabetes for an extra 100g/week of fish intake in the subset of studies adjusting for: a) Family history of diabetes b) Waist circumference c) Fish oil supplements (InterConnect project on the association between fish intake and type 2 diabetes).

		**Men**		
	**Studies (n)**	**IRR (95% CI)**	**p**	***I*²**
**a)**	14			
Model 2		1.00 (0.98, 1.02)	0.73	58%
Model 2 + **Family history of diabetes**		1.00 (0.98, 1.02)	0.69	63%
**b)**	15			
Model 2		1.00 (0.99, 1.02)	0.52	50%
Model 2 + **Waist circumference**		1.00 (0.99, 1.02)	0.45	52%
**c)**	6			
Model 2		1.00 (0.99, 1.01)	0.49	0%
Model 2 + **Fish oil supplements**		0.99 (0.98, 1.01)	0.91	0%
		**Women**		
	**Studies (n)**	**IRR (95% CI)**	**p**	***I*²**
	17			
Model 2		1.01 (0.99, 1.02)	0.07	59%
Model 2 + **Family history of diabetes**		1.01 (0.99, 1.02)	0.1	58%
	20			
Model 2		1.01 (1.00, 1.03)	0.04	63%
Model 2 + **Waist circumference**		1.02 (1.00, 1.03)	0.002	57%
	7			
Model 2		1.00 (0.98, 1.01)	0.77	12%
Model 2 + **Fish oil supplements**		0.99 (0.98, 1.01)	0.91	0%

Model 2 adjusted for age, sex (if applicable), education, smoking, physical activity, alcohol intake, BMI, co-morbidities at baseline, energy intake, fibre, red and processed meat, fruit, vegetables, sugary drinks.

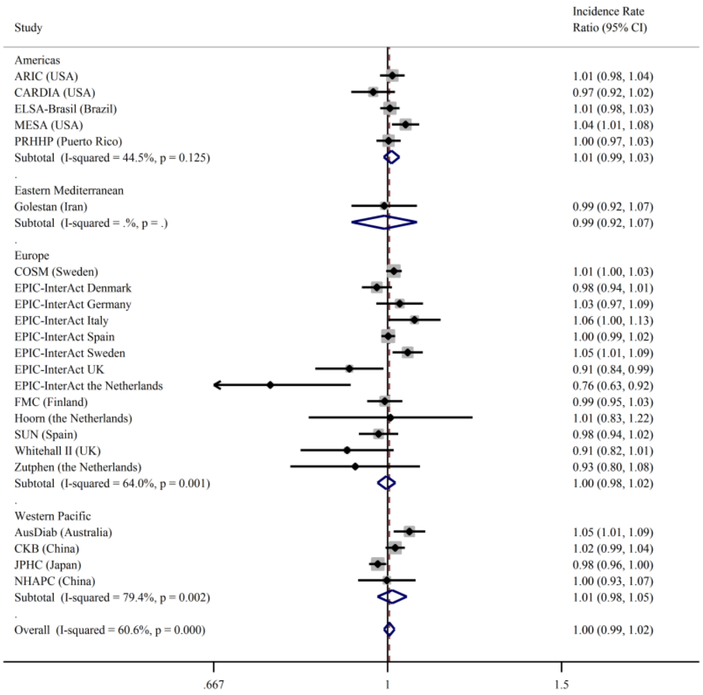

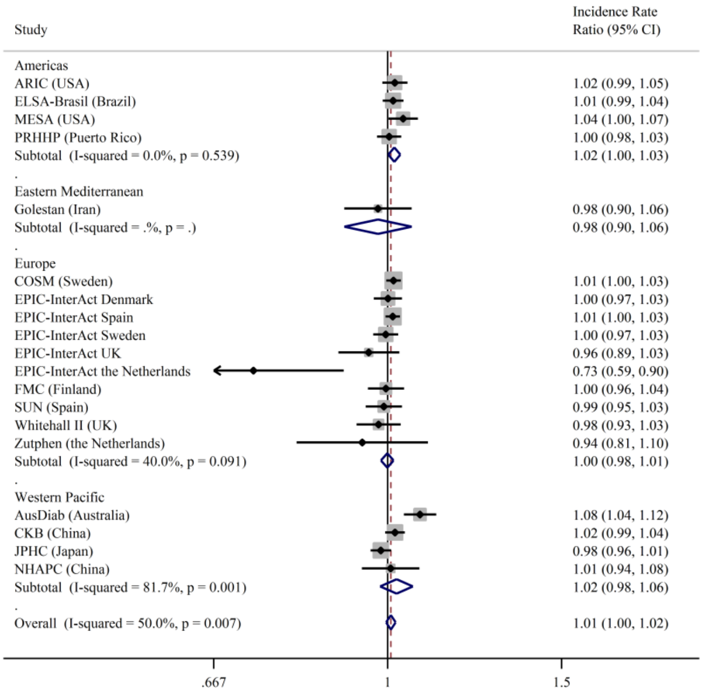

Figure S1. Incidence rate ratios and 95% confidence intervals for the association between the consumption of total fish (per 100g/day) and incident type 2 diabetes (secondary outcome) in InterConnect men. Model 1 (upper panel) adjusted for age, education, smoking, physical activity, alcohol intake, BMI, co-morbidities at baseline. Model 2 (lower panel) was additionally adjusted for dietary factors: energy intake, fibre, red and processed meat, fruit, vegetables, sugary drinks.
ARIC, Atherosclerosis Risk in Communities; ELSA Brasil, Brazilian Longitudinal Study of Adult Health; CARDIA, Coronary Artery Risk Development in Young Adults Study; MESA, Multi-Ethnic Study of Atherosclerosis; PRHHP, Puerto Rico Heart Health Program; FMC, Finnish Mobile Clinic Health Examination Survey; COSM, Cohort of Swedish Men; SUN, Seguimiento Universidad de Navarra (University of Navarra

Follow-up); AusDiab, Australian Diabetes, Obesity and Lifestyle Study; CKB, China Kadoorie Biobank; JPHC, Japan Public Health Center-based; NHAPC, Nutrition and Health of Aging Population of China Study; SMHS, Shanghai Men Health Study.

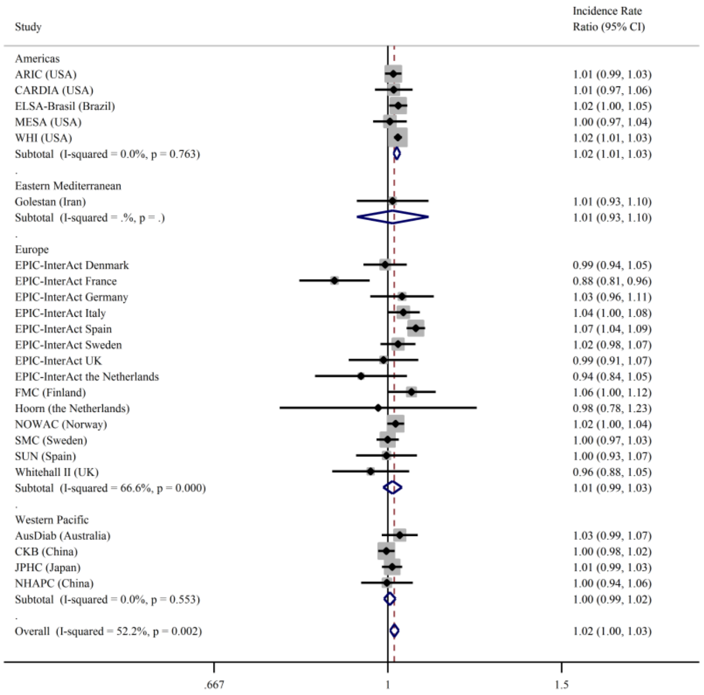

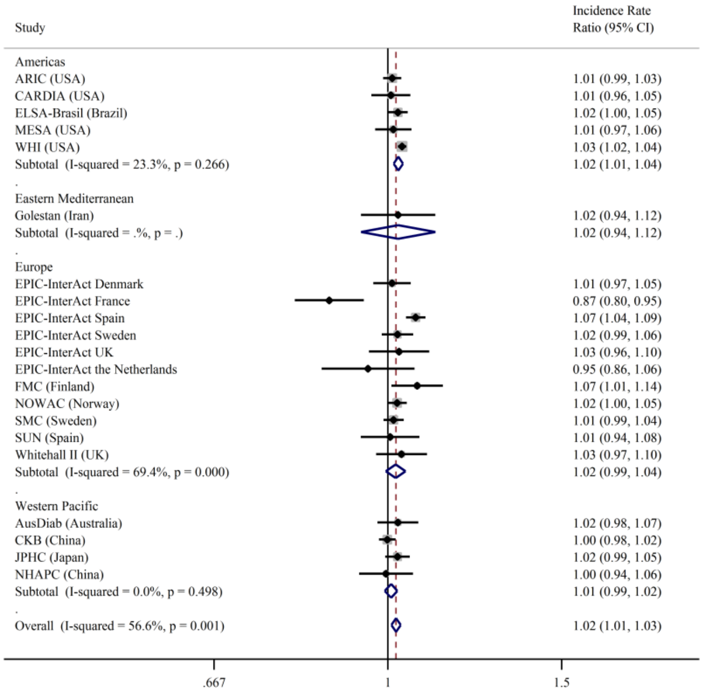

Figure S2. Incidence rate ratios and 95% confidence intervals for the association between the consumption of total fish (per 100g/day) and incident type 2 diabetes (secondary outcome) in InterConnect women. Model 1 (upper panel) adjusted for age, education, smoking, physical activity, alcohol intake, BMI, co-morbidities at baseline. Model 2 (lower panel) was additionally adjusted for dietary factors: energy intake, fibre, red and processed meat, fruit, vegetables, sugary drinks.
ARIC, Atherosclerosis Risk in Communities; CARDIA, Coronary Artery Risk Development in Young Adults Study; ELSA-Brasil, Brazilian Longitudinal Study of Adult Health; MESA, Multi-Ethnic Study of Atherosclerosis; WHI, Women Health Initiative; FMC, Finnish Mobile Clinic Health Examination Survey; NOWAC, Norwegian Women and Cancer; SMC, Swedish Mammography Cohort; SUN, Seguimiento

Universidad de Navarra (University of Navarra Follow-up); AusDiab, Australian Diabetes, Obesity and Lifestyle Study; CKB, China Kadoorie Biobank; JPHC, Japan Public Health Center-based; NHARC, Nutrition and Health of Aging Population of China Study; SWHS, Shanghai Women Health Survey
